# A low-cost, high-performance video-based binocular eye tracker for psychophysical research

**DOI:** 10.16910/jemr.14.3.3

**Published:** 2021-05-05

**Authors:** Daria Ivanchenko, Katharina Rifai, Ziad M. Hafed, Frank Schaeffel

**Affiliations:** Ophthalmic Research Institute, Tübingen, Germany; Carl Zeiss Vision International GmbH, Aalen, Germany; Werner Reichardt Centre for Integrative Neuroscience, Germany; Hertie Institute for Clinical Brain Research, Tübingen, Germany

**Keywords:** Eye movement, eye tracking, saccades, microsaccades, vergence, usability

## Abstract

We describe a high-performance, pupil-based binocular eye tracker that approaches the performance of a well-established commercial system, but at a fraction of the cost. The eye tracker is built from standard hardware components, and its software (written in Visual C++) can be easily implemented. Because of its fast and simple linear calibration scheme, the eye tracker performs best in the central 10 degrees of the visual field. The eye tracker possesses a number of useful features: (1) automated calibration simultaneously in both eyes while subjects fixate four fixation points sequentially on a computer screen, (2) automated realtime continuous analysis of measurement noise, (3) automated blink detection, (4) and realtime analysis of pupil centration artifacts. This last feature is critical because it is known that pupil diameter changes can be erroneously registered by pupil-based trackers as a change in eye position. We evaluated the performance of our system against that of a wellestablished commercial system using simultaneous measurements in 10 participants. We propose our low-cost eye tracker as a promising resource for studies of binocular eye movements.

## Introduction

Eye tracking is becoming increasingly pervasive in many applications: mobile phones, cars, laptops, movies, marketing, education, and video games ( 1, 2, 3, 4, 5). Moreover, eye trackers now find use in rehabilitative and assistive applications (e.g., controlling of wheelchairs, robotic arms, and other prostheses) (6, 7). In research laboratories, eye trackers are now a necessity, if not for anything else, then at least for controlling where subjects look. In fact, even in animal models where eye movements have not been traditionally considered, like mice in visual neuroscience applications, eye tracking is now becoming more commonplace (8, 9, 10). However, with prices reaching a few tens of thousands of dollars, the costs of easy-to-use, non-invasive commercial eye tracking systems can be very prohibitive for research laboratories. This hampers even wider spread use of eye tracking technology for psychophysical research, and particularly in emerging world regions interested in furthering their investments in science (11, 12).

The most frequently available options for eye tracking can generally be divided into two main measurement principles: optical and electromagnetic. Optical eye trackers use real-time video image processing techniques, typically tracking the first Purkinje image (also called the corneal reflection or the glint) (13) and the pupil center. The eye is usually illuminated with infrared light to increase the contrast of the video images without disturbing the subject with visible light. Some other optical techniques also use the first and fourth Purkinje images (so-called “dual Purkinje image eye trackers” (14)). These systems are accurate, but they are harder to implement, especially because of the reduced contrast of the fourth Purkinje image. Temporal resolution in optical approaches is limited by the video frame rate. Spatial resolution is ultimately limited by pixel resolution and pixel noise. In terms of drawbacks, tracking the pupil center relies on the assumption that it changes position only when the eye rotates. However, it is known that when the diameter of the pupil changes, this can result in a “de-centration” of the pupil center even without a concomitant eye movement (15). Further limitations are also that the movements of the pupil center relative to the first Purkinje image may not be linearly related to eye position because the corneal surface curvature is not spherical and the center of rotation of the globe does not coincide with the center of curvature of the cornea (16). If eye position is tracked over a large angular range, multiple fixation points become necessary for calibration as linearity between eye position and the distance between pupil center and first Purkinje image can no longer be assumed (17). If the first and fourth Purkinje images are used for eye tracking, it must also be kept in mind that the crystalline lens is not rigidly attached to the globe, but may exhibit spatial jitter during saccades, called “lens wobble” (18).

Electromagnetic eye trackers use “search coils” ( 19, 20, 21, 22, 23), which are loops of wire that are meant to rotate with the eye. In human subjects, the coils are attached to a contact lens that the subject wears; in animal subjects, the coils are implanted sub-conjunctively around the sclera. In both cases, a wire is led out of the eye to a connector, and that is why this technique is not very popular with human studies (naïve subjects typically require training for use of the coils, and individual sessions are short). With the coils in place, the subject sits head-fixed in magnetic fields that induce a current in the coils. Depending on eye orientation in the magnetic fields, different currents are induced in the search coils. Search coil eye trackers have very high spatial resolution, and they can be digitized at large temporal frequency (typically 1 KHz). A major disadvantage of electromagnetic eye trackers is that they are invasive, while optical eye trackers do not get in touch with the eye.

Due to the price of commercial devices, scientists and engineers have tried many times to build a low-cost, easily available eye tracker. Among the most successful devices are the Eye Tribe (Oculus VR, California, USA) (99$), the GazePoint (GP3) (Vancouver, Canada) (495$), and the Tobii EyeX Tracker (Stockholm, Sweden) (695$). The price of these devices is relatively low in comparison with other trackers from commercial companies, but the problem is that they do not always provide high frequency measurements (typically only reaching up to 60 Hz) or good accuracy and precision ( 24, 25, 26, 27, 28, 29). It was shown that the accuracy of the EyeTribe and GP3 is in the range of 0.5 and 1 degrees (26), and the spatial resolution of EyeTribe is 0.1 degrees (26). Moreover, studies showed that main saccades characteristics derived from EyeTribe data (e.g., saccade amplitudes, durations, and peak velocities) were different from those normally observed in eye movement recordings of healthy participants (29). The most recent low-cost eye tracker that we could find was built by a German laboratory and is called RemoteEye (20). The price of the device is suggested to not exceed 600 euros, and it runs with a frequency of up to 570 Hz monocularly. The eye tracker showed an accuracy of 0.98 degrees and precision of 0.38 degrees.

In this paper, we describe our development of a custom-built video-based eye tracker that is much cheaper than commercial alternatives, but with similar performance (or even better for some benchmarks). We document its performance limits, and how it can be built using standard off-the-shelf components. We also describe how we have incorporated in our software algorithms features that would be very important for some applications, such as the study of fixational eye movements. For example, our eye tracker handles the above-mentioned pupil de-centration issue. An important feature of our eye tracker is that it is fully binocular. This is important because, even though we use binocular viewing in most normal circumstances, a substantial amount of eye tracking research relies only on monocular tracking. We believe that making available a low-cost binocular eye tracker can trigger interesting future investigations of binocular eye movements and stereo vision.

## Methods

### Set-up and hardware

The binocular eye tracker (see Figure 1a) consists of two infrared sensitive monochrome USB3.0 cameras (The Imaging Source, www.theimagingsource.com, camera model DMK33UX174). Both cameras are run at a video frame size of 640x480 pixel and 8-bit grey levels (software selectable monochrome video format: Y800) with a frame rate of 395 Hz (specified maximal frame rate of the cameras and checked by counting the number of frames that were processed in 60 secs). Both cameras are equipped with a lens with 50 mm focal length and a f/# of 1.4 (ROCOH TV Lens 50 mm 1:1.4). The camera sensor specifications are as follows: ^1^/_1.2_-inch Sony CMOS Pregius sensor (IMX174LLJ); pixel size is H: 5.86 µm, V: 5.86 µm. The number of effective pixels is 1936 (H) x 1216 (V), with the maximum resolution being 1920 (H) x 1200 (V). The lenses are covered by a daylight cut-off filter (The Imaging Source, https://www.theimagingsource.de/produkte/optik/filter/, #092, 46 x 0.75). Three 5 mm extension rings are necessary to focus the cameras on the eyes at a distance of 250 mm which results in a video magnification of 39.7 pixel/mm. Both eyes are illuminated by a single circular arrangement with a diameter of 40 mm of 15 high power IR LEDs emitting at 875 nm (https://www.conrad.de/de/p/hp-hdsl-4230-hp-ir-emitter-875-nm-17-5-mm-radial-bedrahtet-185809.html). The LED field was placed 85 mm below the cameras and adjusted to illuminate both eyes from below and generate two bright and large Purkinje images in the two eyes. We used a gaming computer (Memory PC Intel i7-7700K 4X 4.2 GHz, 4 GB DDR4, 500 GB Sata3) and a computer screen with a refresh rate of 240 Hz (Acer Predator XB252Q, 24.5”, resolution of 1920 x 1080 pixels) (see Figure 1b), although neither is mandatory to do binocular eye tracking at the full speed of 395 Hz.

**Figure 1.**
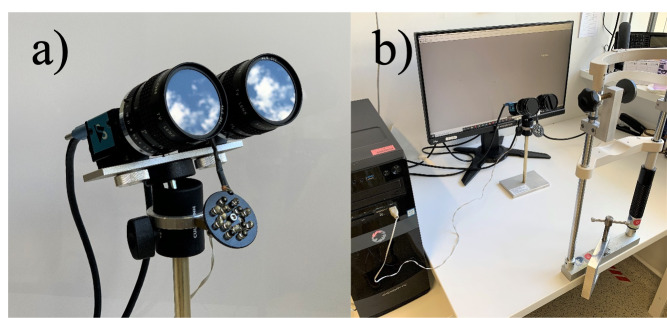
(a) Our custom-built binocular eye tracker with two infrared cameras and LED array below them. (b) Our eye tracker set-up consisting of the eye tracker by itself, the gaming computer, the computer screen, and a chin rest. Note that for studies on binocular interactions, we are primarily interested in eye movements within a range of approximately +/- 5 deg from the center of the screen; thus, occlusion of part of the screen by our eye tracker cameras is not problematic.

### Software and estimated achievable spatial resolution

Software was developed under Visual C++ 8.0 to merge both camera inputs into one video buffer and to track both pupil centers and first Purkinje images (see Figure 2). Bright and large first Purkinje images were generated by the circular field of 15 infrared LEDs below the cameras. It can be simply estimated how precisely the center of the pupil and the first Purkinje image position must be determined to achieve an angular resolution of 1 arcmin. It is known (17, 30) that, on average, the first Purkinje image moves one millimeter relative to the pupil center when the eye rotates about 12 degrees (Hirschberg ratio). Accordingly, for one degree, the displacement would be 83 µm; for 1 arcmin of eye rotation, it would only be 1.39 µm – close to one thousandth of a millimeter. This estimation illustrates how precisely the pupil center and first Purkinje image center need to be detected to reliably measure fixational eye movements, for example. Pixel magnification in the current set-up was 39.7 pixel/mm or 25.2 µm/pixel. Accordingly, a one-pixel change in position in pupil center of the first Purkinje image was equivalent to 18.1 arcmin, not yet the range of fixational eye movements. However, because a 4 mm pupil already generates about 20,000 dark pixels and a bright first Purkinje image about 400 pixels, their centers of mass could be determined with subpixel resolution for their positions. In our setup, the positions were determined with a resolution of 0.2 pixels, equivalent to about 3.6 arcmin. The pupil was located by a simple thresholding procedure – all pixels that were darker than an adjustable threshold (default: 0.6 darker than the average image brightness) were stored, the center of mass determined, and the pupil area measured as the number of dark pixels. Pupil radius was determined as

(1) $$\text{r = }\;\sqrt{\frac{\text{number of pixels}}{\pi }}$$

The pupil border was graphically denoted by a circle and could be optimized by manually adjusting the threshold. The same procedure with an inverted threshold was applied to determine the center and diameter of the first Purkinje image, which was also marked with a green circle. The pixels in the Purkinje image are typically close to saturation, and the pixel threshold for their detection was set to 250, independently from the average brightness of the video image. That is, pixels higher than 250 in intensity were considered part of the Purkinje image. We have also included a simple focus detection algorithm, counting the number of pixels in the Purkinje image. The size of the Purkinje image is determined by the size and distance of the IR LED field that generates it, and also of defocus.

**Figure 2.**
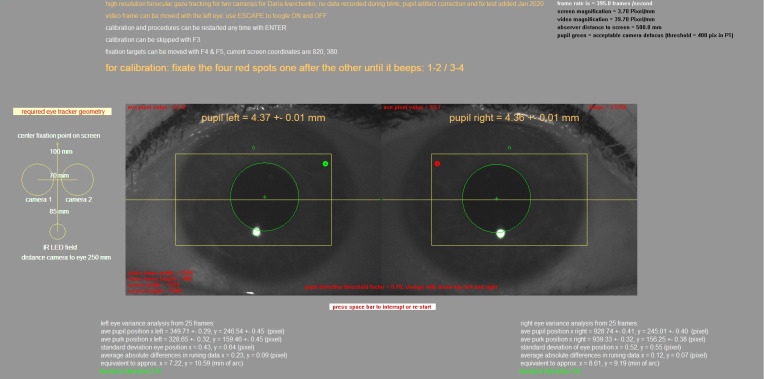
Screenshot of the eye tracker software output, also showing the raw video images from both cameras. Large green circles mark the pupil borders, and small green circles (when visible) show the borders of the first Purkinje images. The yellow rectangles denote the areas in which the pupil is detected. The pixel coordinates of the pupil center, the pixel coordinates of the center of the first Purkinje images, and the standard deviations from 25 measurements are continuously displayed in the bottom. Note that standard deviations are below one pixel. Standard deviations are also provided in minutes of arc.

We used the PC-CR (pupil center – corneal reflection) vector technique to measure the angular position of the eyes (31). The detected eye positions are initially written down to the file in pixel coordinates (the coordinate system of the image), but we used the calibration procedures described below to also obtain degrees of visual angle.

### Real-time noise analysis

To determine how stable the detection of the pupil center and the center of the first Purkinje image was, a running standard deviation was determined, continuously taking the data of the latest 25 samples. Sampling at 395 Hz, 25 data points are equivalent to 63 ms, which is too short to be severely affected by ocular drifts. It therefore reflects how repeatably the positions are detected in each frame. Standard deviations ranged from 0.2 to 0.5 pixels. These data are continuously displayed on the screen for both eyes to be able to judge the reliability of eye tracking. In addition, a more conservative measure of measurement noise was performed – determining the average absolute difference between two subsequent measurements in the horizontal direction, again determined over the latest 25 measurements. These data were also displayed.

Since the standard deviations of pupil sizes over the latest 25 frames were also available, they could be used as a sensitive way to detect blink artifacts. During blinks, the pupil is rapidly covered by eye lids and the number of black pixels declines. A standard deviation of pupil sizes exceeding 0.2 mm was found to denote blinks (since pupil size cannot change fast, and pupil responses are slow in the absence of blinks). In this case, data were continuously written, but the data file contained zeros in all data columns.

### Calibration procedure

Because the same LED field served as a light source for both eyes, the first Purkinje images were not at the same position in the pupils of both eyes, and a calibration procedure was done simultaneously for both eyes, but independently. Four red fixation points (diameter 4 arcmin) appeared on the screen, one after the other. They were arranged in a rectangle, which could be adjusted in size from the keyboard before calibration. When the subject fixated, the running standard deviation of eye positions dropped to a value below 0.5 degrees. This triggered the fixation point to turn green, and the averages of 100 samples and 100 first Purkinje images were stored. The next fixation point appeared, and the procedure was repeated. After the calibration procedure was completed (i.e. after approximately 2-3 seconds), any eye position within the rectangular field could be inferred by linear extrapolation. At this point, it is necessary to consider how linearly the distance between pupil center and Purkinje image center are related to the true eye position. Linearity of this procedure was tested for the central +20 degrees (display size of approximately +20 cm from the screen center) of the visual field in the experiments described below. Outside this range, irregularities of corneal curvature as well as changes in the position of the center of rotation of the eyeball cause non-linear conversions into eye positions, which were not analyzed for the present paper. More sophisticated calibration procedures can account for such non-linearities, depending on the intended application of the eye tracker (17).

### Effects of pupil size on pupil center positions

Since a stationary pupil center position cannot be assumed when pupil size changes (15), we implemented an automatic procedure to correct for potential pupil center drifts when measuring binocular fixational eye positions. These binocular fixation measurements are the measurements for which pupil center drifts caused by pupil size changes are the most problematic, given the similar amplitudes of the movements and the pupil size artifacts. After calibration, a single fixation point was presented on a black background in the center of the screen for 4 seconds. Due to the black screen, the pupils dilated. While fixation was maintained, the screen suddenly turned brighter to a pixel grey level of 150 (on an 8-bit gray scale) for a duration of 30 frames (about 75 ms), which elicited a prominent pupil constriction. While such a manipulation can also alter fixational eye position (32), the effect on eye position is minute in comparison to pupil center drifts and also occurs before pupil dilation. Eye positions were continuously recorded during the pupil responses. After another 600 ms, the angle of eye convergence was automatically plotted against pupil size. If the pupil center position was not stationary but rather moved when pupil size changed, then this became evident as a significant convergence of eye position. Specifically, the software plotted pupil sizes versus convergence measures and performed a linear regression. If the slope of the regression was significantly different from zero, a correction was necessary, and it was implemented in the subsequently recorded data. This was done by simply taking the regression equation in the plot of vergence versus pupil size, and re-calculating vergence for each of the known pupil sizes.

### Automated tests of gaze accuracy and gaze precision, and comparisons to the commercial EyeLink system

To make sure that our device can measure eye movements and fixations correctly, we compared it to the well-known eye tracker, EyeLink 1000 Plus (SR Research, Ottawa, Ontario, Canada). This is one of the most popular and established commercial devices used for binocular measurements. We built a set-up that included the two eye tracking systems simultaneously: the first one was our computer (Memory PC Intel i7-7700K 4X 4.2 GHz, 4 GB DDR4, 500 GB Sata3), the monitor (Acer Predator XB252Q, 24.5”, resolution of 1920 x 1080 pixels), and the custom-built device, and the second one was the EyeLink 1000 Plus system with its own computer. Stimuli and calibration points were presented on our monitor. We used the chin rest to fix participants’ head in order to avoid any unnecessary movements. The calibration procedure included four points appearing on the screen in sequence. The EyeLink 1000 Plus (desktop mode, binocular, 25 mm lens, stabilized head) was recording data without calibration; we calibrated it later offline using the same calibration points as those used for the custom-built eye tracker. To compare the temporal and spatial performance of our eye tracker with an established device (EyeLink 1000 Plus), a TTL (transistor-transistor logic) signal was generated by the custom-built eye tracker each time a new fixation target appeared. This signal was fed into the EyeLink system as an external event (similar to button press devices connected to the EyeLink system). This served as a time stamp for simultaneous data recording with both devices (see Figure 3). We used the infrared illuminator (LED) from the custom-built eye tracker for both devices. This was acceptable because the spectral content of our illuminator was very similar to that of the EyeLink system (as we confirmed experimentally by measuring them). Before the experiment started, we made sure that the eye was perfectly illuminated in the EyeLink eye tracker. This allowed us to make simultaneous recordings.

**Figure 3.**
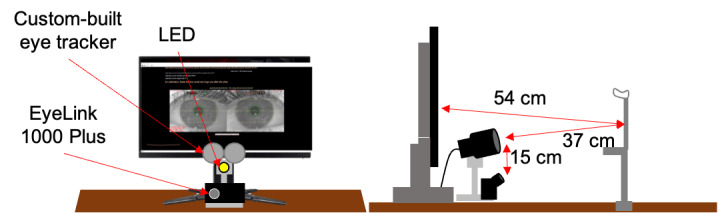
Illustration of our experimental set-up for comparing the performance of our eye tracker to that of a commercial system (EyeLink 1000 Plus). Left: view from the position of the participant showing the eye tracker’s screen. LED refers to the circular field of 15 IR LEDs that generated the first Purkinje images used for eye tracking. Right: side view illustrating the distances between the participants’ eyes, the screen, and the two eye-trackers.

### Data recording

After the recording session, the following data could be written to a file for each single frame: computer time, frame number, pupil sizes for left and right eyes (mm), x positions for left and right eyes in screen coordinates of the fixation points (pixel), vergence in arcmin with and without pupil centration correction, x and y positions for fixation targets, “1” when a TTL signal was emitted and “0” if there was none.

### Participants

We measured ten participants (three male, age range 21–26 years). They had no known ocular pathologies or binocular irregularities, other than moderate refractive errors that were corrected by their habitual spectacles or contact lenses. The experiment was conducted in agreement with the Code of Ethics of the World Medical Association (Declaration of Helsinki) and approved by the Ethics Commission of the University of Tuebingen. Informed consent was obtained from all participants.

### Measurements using artificial eyes

The only way to completely eliminate any eye movements and other biological factors from the eye tracker signal is to use artificial eyes (33). For better and more optimal comparison of the precision between our eye tracker and the commercial system, we first used the artificial eyes shown in Figure 4 (MagiDeal, 16 mm, https://www.amazon.de/St%C3%BCck-H%C3%A4lfte-Acryl-Dollfie-Eyeballs/dp/B008S3S9H2). These artificial eyes were very similar to real ones since they also had an iris, cornea, and even a corneal reflection.

**Figure 4.**
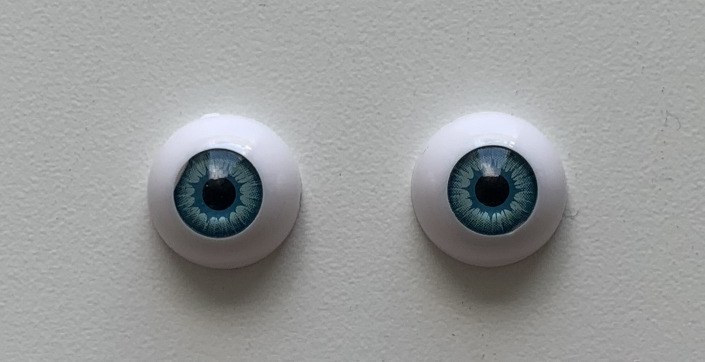
The artificial eyes that we used for precision analyses across eye trackers.

The eyes were made of acrylic, and they had a diameter of 16 mm. Pupil diameter was 4 mm. The eyes were mounted on the chin rest at the same distance and height as the participants’ eyes, and we proceeded to perform simultaneous measurements with both eye trackers. We avoided desk vibrations as much as possible, to avoid measuring artifactual displacements.

### Binocular vergence eye movement measurements

In order to demonstrate that our eye tracker was well prepared for doing binocular measurements, we performed an additional experiment exercising vergence eye movements. We asked a participant to look at three different targets located at different distances from the computer screen while measuring eye movements with our eye tracker. We used the same calibration procedure as described above before asking the participant to look at the different distances.

For each trial we used two targets between which the participant fixated. One target was located on the computer screen, and the other one was located on a special holder similar to the one that we used to hold the cameras of our eye tracker. The holder was mounted on a metal horizontal panel. This panel allowed us to move the target back and forth depending on the distance of the target that we wanted to apply. Both targets were 1x1 mm yellow squares. They were created using yellow tape.

The monitor was located at 54 cm from the participant’s eyes. We first put one target at a distance of 49 cm from participant’s eyes. Next, the target was located at 44 cm, and then the last one was at a distance of 29 cm. During the first trial, the participant was asked to look first at the target located on the screen (this corresponded to 6.360 degrees of vergence angle), and then to the target located at 49 cm (7.008 degrees) from the eyes. The next task was to look at the first target (6.360 degrees) and then to the target located at a distance of 44 cm (7.800 degrees). During the last trial, the participant was looking at the target with the distance of 54 cm (6.360 degrees) and then at 29 cm (11.812 degrees).

### Data analysis

For the offline calibration of the EyeLink 1000 Plus system, we first chose fixation periods (free of saccades and microsaccades) of 100 ms for each calibration point (similar to our calibration approach of our custom-built eye tracker). After that, the average eye position of this piece of data was found. For each of the five calibration points (including the center point), we obtained a best-fit second-order polynomial for the measurements (34, 35).

Saccades and microsaccades were detected using U’n’Eye – a deep neural network for the detection of saccades and other eye movements (36). First, we trained the network on our data. For this, we took 60 seconds of data that included fixations, saccades, and microsaccades. For the training set, saccades and microsaccades were manually labeled with 1 and fixations with 0. The output of the training process was the trained network weights that were later used for the saccade detection. In the end, we had a file with saccade labels for each trial.

## Results

### Precision and accuracy using artificial eyes

An eye tracker performance is usually described using two metrics: precision and accuracy. Precision is the ability of the eye tracker to reliably reproduce the same gaze point measurement (36). Precision values of currently available pupil-based eye trackers range from 0.01 to 1 degree (36). Accuracy is the average difference between the real stimulus position and the measured gaze position. Typical accuracy values for pupil-based eye trackers fall in a range between 0.5 and 1 degrees (36). Accuracy and precision are usually measured separately for horizontal and vertical positions, and for the right and the left eye, or as an average of both eyes.

We estimated the precision of our binocular eye tracker using two methods: 1) by calculating the horizontal and vertical root mean square (RMS) noise (that is: the RMS of the inter-sample angular distances) over all samples, and 2) by calculating the horizontal and vertical standard deviation of the samples. The RMS noise was calculated using the following equation

(2) $$RMS=\;\sqrt{\frac{1}{n}\sum_{i=1}^{n}\theta_{i}^{2}}=\;\sqrt{\frac{\theta_{1}^{2}+\;\theta_{2}^{2}+\ldots +\theta_{n}^{2}\;}{n}}$$

where *θ* means the angular distance between successive fixation data samples (*x* *_i_*, *y* _*i* _) to (*x* _*i* + 1_, *y* _*i* + 1_) (sample-to-sample distances). The resulting values were averaged across trials.

For the best comparison of precision between devices, we used artificial eyes. The measurements took place under the same light conditions for both eye trackers. We used the same methods of calculating precision RMS and precision standard deviation as described above. One trial of recording the data took 15 seconds, and we later divided the measurements into one-second epochs. Precision was calculated across 15 epochs and then averaged across them. The results are summarized in Table 1 for both our eye tracker as well as the EyeLink 1000 Plus system. As can be seen, our eye tracker outperformed the much more expensive system for horizontal eye movements, and it exhibited similar performance for vertical eye movements. This is despite the fact that our eye tracker had a lower sampling rate. However, this is not a major issue given the bandwidth of eye movements in general and given that precision standard deviation measures are less dependent on the sampling rate of the eye tracker (36).

**Table 1.** Precision RMS and standard deviation, both in degrees of visual angle. Data were obtained from the custom-built eye tracker and an EyeLink 1000 Plus, using artificial eyes. The numbers in parentheses indicate standard deviation of the measurements across 15 repetitions.

	Custom-built eye tracker	EyeLink 1000 Plus
Precision (RMS)		
horizontal	0.0353 (0.0028)	0.0406 (0.0091)
vertical	0.003 (1.6092e-04)	0.0032 (1.3606e-04)
Precision (standard deviation)		
horizontal	0.0252 (0.0018)	0.0361 (0.0062)
vertical	0.0061 (3.2828e-04)	0.0074 (0.0022)

### Raw data plots (human participants)

**Figure 5.**
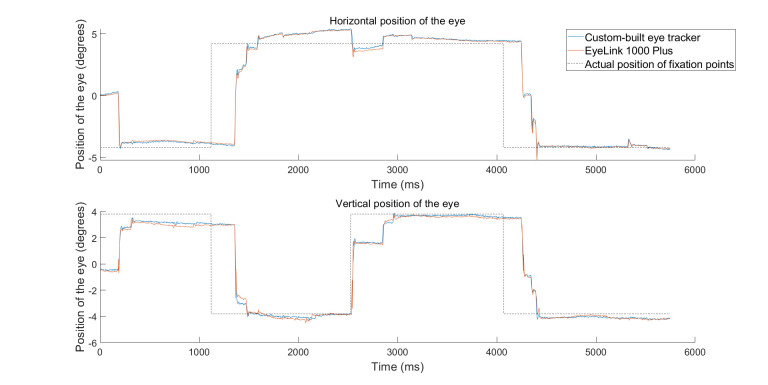
Raw data plots showing horizontal and vertical positions of the left eye for the custom-built eye tracker and EyeLink 1000 Plus. Blue line – custom-built eye tracker; orange line – EyeLink 1000 Plus, dashed black line – the actual position of the fixation point at the time of the experiment. Both eye trackers largely agreed, but there were subtle differences in reported fixation position. Subsequent figures explore the reasons for such subtle differences.

Having established the robustness of our eye tracker with artificial eyes, we next aimed to validate its performance with real data obtained from human participants. We recruited a total of ten participants who performed simple fixation and saccade tasks. Figure 5 shows raw data plots obtained from one sample participant. The curves in blue show the measurements of eye position with our custom-built binocular eye tracker.

The curves in orange show the measurements with the EyeLink 1000 Plus system. The participant was asked to track the position of a fixation spot as it jumped on the display (fixation spot locations are shown in the figure with dashed black lines; note that there is a delay between fixation spot jump and reactive saccade due to physiological reaction times). For simplicity, we show only the positions of the left eye, but the measurements were naturally binocular. As can be seen, simultaneous measurements of eye position between the two systems largely overlapped. In particular, saccade times were coincident. However, there were also subtle differences in eye position reports in some cases. Our summary analyses below explain the possible reasons for such discrepancies.

### Precision and accuracy with participants

**Figure 6.**
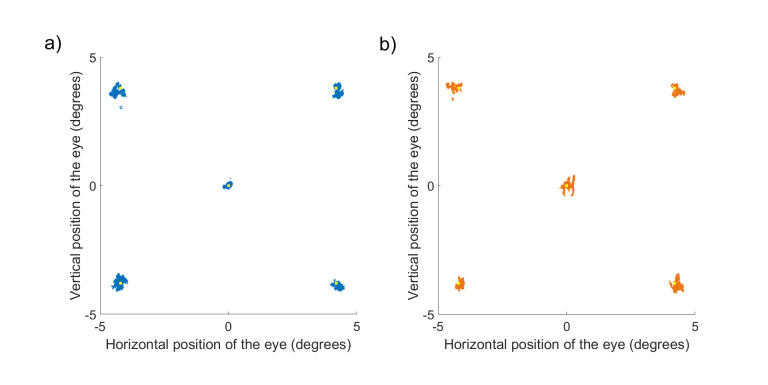
All samples (every eye tracker sample that was obtained during the experiment excluding saccades and blinks) obtained from one participant using our custom-built eye tracker (a, blue dots) and the EyeLink 1000 Plus (b, orange dots). The experiment consisted of presenting five single targets at five different spatial locations (1500 milliseconds each). Yellow squares indicate true target locations. Note that some portion of the variability in the shown data is due to physiological drifts in eye position during fixation.

Across participants, we obtained an accuracy estimate by picking a fixation interval in a given trial and averaging horizontal and vertical eye position during this interval. The intervals included periods of time when participants were fixating the certain target excluding saccades, microsaccades, and blinks. During these intervals, participants were given an instruction to fixate the target for 1.5 seconds. Figure 6 shows example measurements from one participant for all five fixation points. As can be seen, both eye trackers performed well, but the error between target and eye positions in the EyeLink 1000 Plus system was bigger. To quantify this, we calculated a horizontal or vertical average offset within a trial from the true target location. We did this for each participant after excluding missing data, blinks, and microsaccades. All precision and accuracy calculations were done using the data obtained from the left eye of each participant. The resulting values were averaged across all participants. For precision, we used similar procedures to those described above with artificial eyes.

For the participant in Figure 6 (the same person as that shown in Figure 5), the average eye position error with our eye tracker was 0.4304 degrees, whereas it was 0.7848 degrees with the EyeLink 1000 Plus system. Thus, our eye tracker outperformed the EyeLink 1000 Plus system.

Across participants, Table 2 provides quantitative numbers.

**Table 2.** Accuracy (mean difference between target location and observed point in degrees of visual angle) and precision (RMS noise and standard deviation in degrees of visual angle) with real data. Data were obtained from the custom-built eye tracker and an EyeLink 1000 Plus system, using five fixation targets (see Figure 6), with a viewing time of 1.5 seconds each. Fixation points spanned a range from -4.2 to 4.2 degrees horizontally, and -3.8 to 3.8 vertically around the display center. The numbers in parentheses denote standard deviations across repeated measurements.

	Custom-built eye tracker	EyeLink 1000 Plus
Precision (RMS)		
horizontal	0.0457 (0.0301)	0.0202 (0.0297)
vertical	0.0467 (0.0310)	0.0271 (0.0403)
Precision (standard deviation)		
horizontal	0.1953 (0.1861)	0.1746 (0.1972)
vertical	0.1984 (0.1812)	0.2160 (0.1944)
Accuracy		
horizontal	0.3858 (0.2488)	0.5504 (0.3051)
vertical	0.4750 (0.4718)	1.0192 (0.7170)

The figure and table also provide our precision estimates. We found that accuracy was better with our eye tracker when compared to the EyeLink 1000 Plus system, but precision RMS was worse. This is explained in part by the higher sampling rate of the EyeLink 1000 Plus system. On the other hand, the superior accuracy performance of our eye tracker is likely due to a much more optimal placement of the cameras – almost level with the participants’ eyes (see Discussion).

### Saccade and microsaccade metrics

We next measured saccade metrics. We detected saccades in the recorded traces independently for our eye tracker and for the EyeLink 1000 Plus system. For this purpose, we used a machine learning approach (37), and we trained a neural network on each eye tracker’s data individually. We then ran the network on the rest of the data.

**Figure 7.**
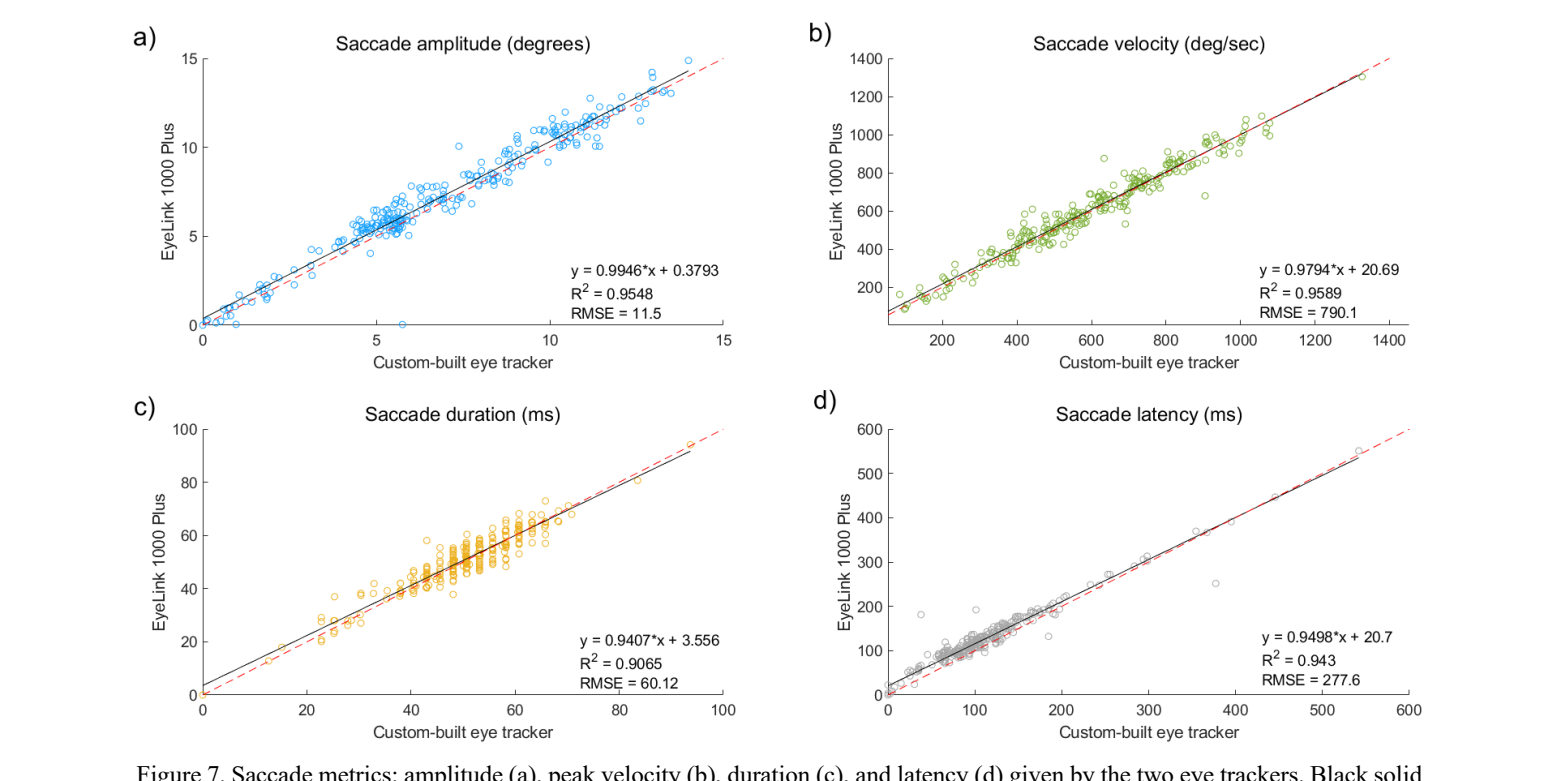
Saccade metrics: amplitude (a), peak velocity (b), duration (c), and latency (d) given by the two eye trackers. Black solid line – regression line; red dashed line – unity slope (all points that have identical X and Y values).

We measured saccade latency, saccade duration, saccade amplitude, and saccade peak velocity. Saccade latency (ms) was defined as the difference between time of the fixation point appearance and the time when saccade happened. Saccade duration (ms) is the time passed from the first point of a saccade to the last one. Saccade amplitude (degrees) was defined as the Euclidean distance between the start point of the saccade and the last point. Peak velocity (degrees/second) was defined as the maximum value in the radial velocity trace. Correlations between the metrics given by the custom-built eye tracker and the metrics given by EyeLink 1000 Plus system were obtained. The results are shown in Figure 7. As can be seen, saccadic metrics were highly correlated in two eye trackers. Although, sometimes small differences in saccade latency and duration existed.

**Figure 8.**
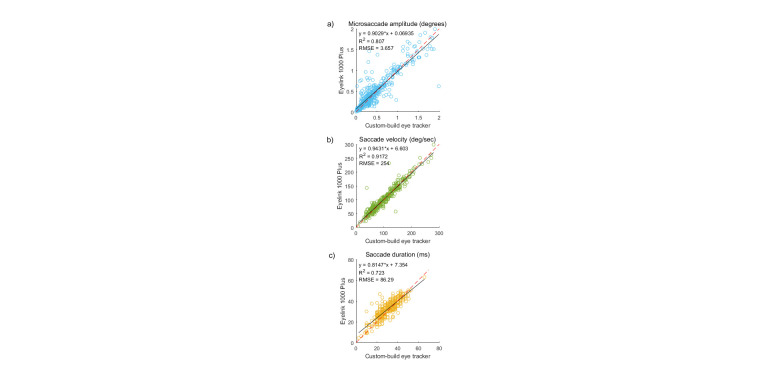
Microsaccade metrics: amplitude (a), peak velocity (b), and duration (c) given by the two eye trackers. Black solid line – regression line; red dashed line – unity slope (all points that have identical X and Y values).

Since fixational eye movements are also of interest in a wide array of psychophysical applications ( 38, 39, 40, 41, 42, 35, 43), we also evaluated how well our eye tracker can detect microsaccades. Both eye trackers could detect microsaccades well. Agreement between the eye trackers was assessed using analyses like those shown in Figure 8.

It is clear from the results that our eye tracker was able to measure microsaccadic metrics. However, some discrepancies existed between the two eye trackers for some saccadometry measures. For example, it can be seen that the correlation between microsaccade amplitude was not as perfect as it was for larger saccades. However, all other parameters, such as duration and velocity, showed a very high statistically significant correlation between the two eye trackers.

We also checked whether our eye tracker missed some microsaccades that were detected by the EyeLink 1000 Plus system, or vice versa. To do this, we took all microsaccades detected by one eye tracker, and we asked what fraction of them was also detected by the other. For all microsaccades detected by our eye tracker, 100% were also detected by the EyeLink 1000 Plus system. However, for all microsaccades detected by the EyeLink 1000 Plus system, 92% of them were detected by ours. This is likely attributed to the lower precision performance of our eye tracker with the real eyes, perhaps due to the lower sampling rate.

### Binocular measurements

Finally, in order to demonstrate that our eye tracker was well prepared for doing binocular measurements, we performed an additional experiment exercising vergence eye movements. We asked a single participant from our lab to look at three different targets located at different distances from the computer screen while we measuring eye movements with our eye tracker.

**Figure 9.**
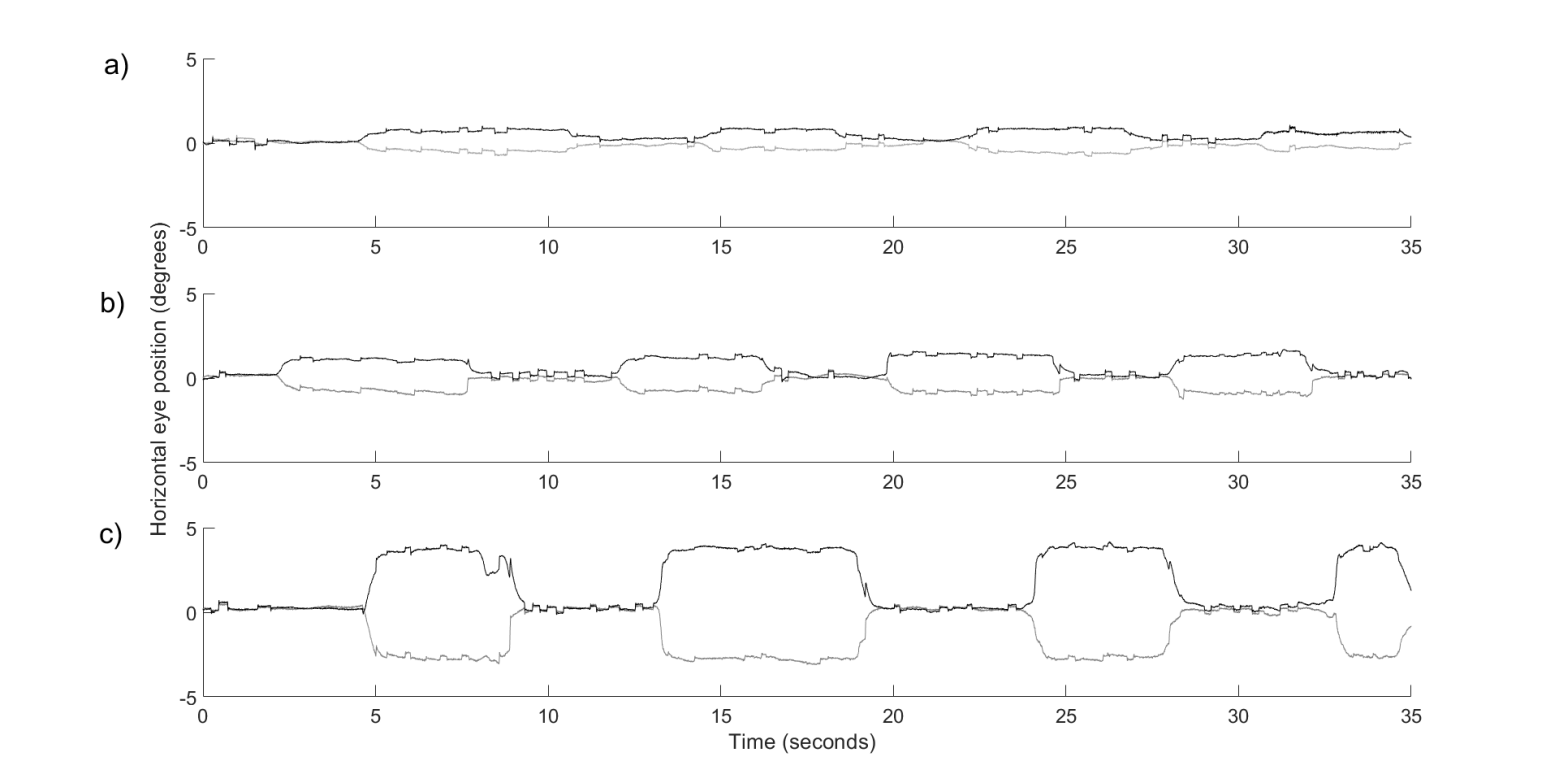
Vergence eye movements shown in one participant while he was asked to look at different targets. (a) The participant was looking at the targets located at a distance of 540 mm (6.360 deg) and 490 mm (7.008 deg) from the eyes. (b) The targets were now at 540 mm (6.360 deg) and 440 mm (7.800 deg) from the eyes. (c) The targets were at 540 mm (6.360 deg) and 290 mm (11.812 deg) from the eyes. Black line – left eye; grey line – right eye of the participant.

The participant was asked to look at two targets in sequence: the first one was always the target located on the screen, and then the other one was located on the holder that was nearer to the participant’s eyes. This induced vergence eye movements that are shown in Figure 9. The subject then alternated back and forth between the target depths. As can be seen, our eye tracker was capable of tracking both small and large convergence and divergence eye movements. This means that our eye tracker is suitable for a wide range of experiments involving binocular vision.

## Discussion

In this article, we introduced an ultra-low-cost custom-built binocular eye tracker. We measured and described its spatial and temporal resolution, as well the limitations of the video image processing algorithms. We also presented a couple of new features that our eye tracker is able to do. These are automatic correction of pupil artifacts and automatic noise analysis. We also compared our eye tracker to the well-known and established EyeLink 1000 Plus (Table 3).

**Table 3.** Comparison table of eye trackers’ characteristics.

Characteristics	Custom-built eye tracker	EyeLink 1000 Plus
Spatial precision (artificial eyes)	0.0191	0.0219
Spatial precision (participants)	0.0462	0.0236
Spatial accuracy	0.4304	0.7848
Sampling rate	395 Hz	1 kHz (binocular), 2 kHz (monocular)
Real-time automated noise analysis	yes	no
Real-time pupil artifact correction	yes	no
Gaze-contingent experiments	yes	no

Our eye tracker’s accuracy and precision were very good under optimal conditions (limited oculomotor range, testing with a chin rest, using the PC-CR approach that improves tolerance to subtle head movements, and using a daylight cut-off filter in a well-lit room), and sufficient to do eye movement research. We found that in comparison with the EyeLink 1000 Plus system, our eye tracker had slightly worse precision but significantly better accuracy. The difference in precision can be explained by the sampling rates of the eye trackers: 395 Hz against 1 kHz. Better accuracy of our eye tracker can be caused by the more beneficial position of our tracker in relation to the participant’s eyes. The EyeLink 1000 Plus is located much lower than the head of a participant, while the cameras of our eye tracker are located on the almost same level with the eyes.

Since in our studies, we are mostly interested in binocular measurements for forward looking with a limited oculomotor range, including small saccades, within a range of +/- 5 deg from the center of the screen, occlusion of part of the screen by our eye tracker cameras is not seriously affecting our measurements.

Simultaneous recording with the custom-built eye tracker and EyeLink 1000 Plus allowed us to compare not only the precision and accuracy of the eye trackers, but also the metrics of saccades and microsaccades. For the measured parameters (amplitude, peak velocity, duration, and latency), we found a high correlation (R>0.9 on average) between the two devices. Microsaccade detection ability is critical to fixational eye movement studies, and here, we showed that microsaccade detection was comparable for the custom-built eye tracker and the EyeLink 1000 Plus system. We suggest that, since our eye tracker has real-time pupil artifact correction, it is also suited for recording and further analysis of drifts. Besides that, our eye tracker is suitable for binocular studies.

In comparison with other low-cost eye trackers, such as the EyeTribe or the PG3, our device has higher frequency (395 Hz against 30 or 60 Hz), which gives scientists the opportunity not only to measure basic saccades and fixations, but to also study smaller eye movements. Our eye tracker has higher precision (EyeRemote – 0.38 degrees, Tobii T60XL Eye Tracker – 0.16 degrees, EyeTribe – 0.1 degrees) and better accuracy (EyeRemote – 0.98 degrees, Tobii T60XL Eye Tracker – 1.27 degrees, EyeTribe and PG3 – from 0.5 to 1 degrees) ( 24, 25, 44, 26, 27, 28, 29). Besides that, our eye tracker is fully binocular, which is very important for some psychophysical experiments.

Another advantage of our eye tracker is that every detail or feature can be easily changed according to the experimental needs. In comparison with denied access to the settings of the EyeLink 1000 Plus system, the detailed description that we provide in Appendix allows researchers to delete or add any eye tracker characteristics as well as change the hardware properties, such as the LEDs or location of the cameras relative to participants’ eyes. It also gives the opportunity to customize the whole experimental set-up in the most convenient way. We are also providing the executable program of our eye tracker software to readers, who can then pair it with their hardware.

Another interesting point to note about eye tracker is that we do not use any smoothing filters in our software. This is potentially very important for studying fixational eye movements, since it was recently shown that some filters could alter the spectral content of measured fixation signals, and therefore give rise to tracker measurements that might appear as natural fixational eye movements (45). We are aware that unfiltered data will cause more noise, but the performance of our eye tracker with artificial eyes showed similar characteristics to the EyeLink 1000 Plus system. This might suggest that, combined with the lack of filtering, our eye tracker may indeed be attractive for the study of fixational eye movements, at least to a similar extent to which the EyeLink 1000 Plus system may be considered attractive for such movements.

In conclusion, we consider our ultra-low-cost eye tracker a promising resource for studies of binocular eye movements as well as fixational eye movements.

### Ethics and Conflict of Interest

The author(s) declare(s) that the contents of the article are in agreement with the ethics described in http://biblio.unibe.ch/portale/elibrary/BOP/jemr/ethics.html and that there is no conflict of interest regarding the publication of this paper.

### Acknowledgements

This study was supported by the Deutsche Forschungsgemeinschaft (DFG, German Research Foundation), project number 276693517, SFB 1233, project TP11.

We are grateful to Dr. Torsten Strasser for his help to merge both camera inputs into one video buffer in Visual C++, and to our participants for their patience and efforts.

## Figures and Tables

**Figure 1A.**
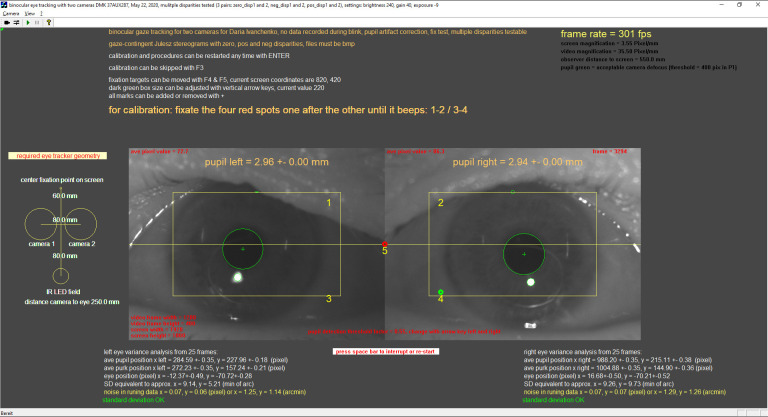
Starting page of the software that shows the “noise” of the eye tracker and starts the calibration.

**Figure 2A.**
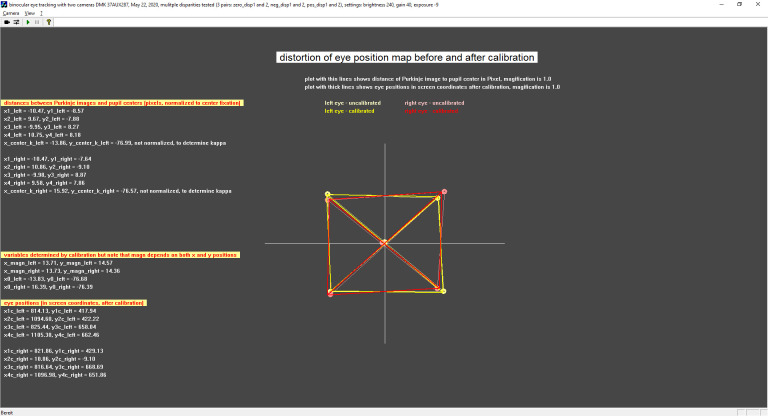
Test of the orthogonality of the calibration, raw data in red, after calibration in yellow.

**Figure 3A.**
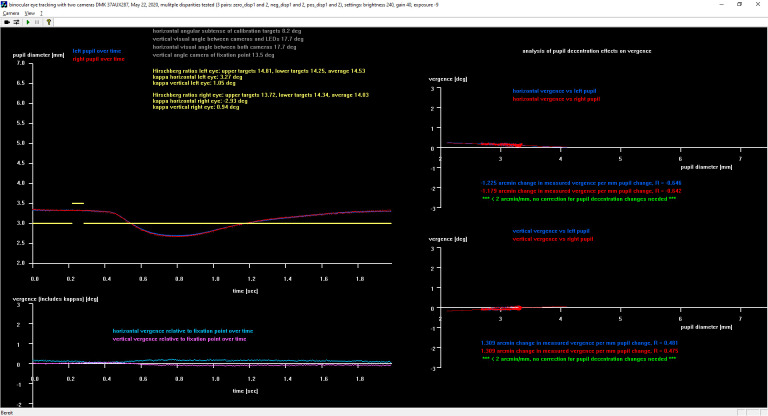
Testing for pupil centration artifacts. Left: A pupil response is elicited by making the screen bright for about 50 msec. Right: The measured convergence of both eyes is plotted against pupil size. If pupil centration would change, a linear fit through the data would have a regression different from zero. Here, the regressions were close to zero which mean that no pupil size dependent correction was necessary for the eye tracker.

**Figure 4A.**
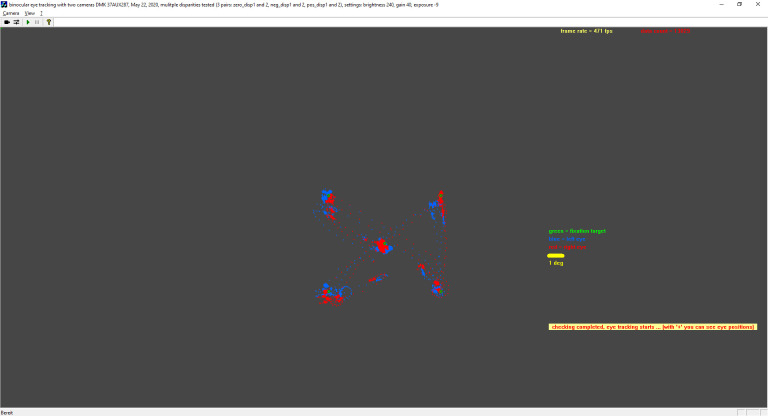
Eye positions when the subject fixated 5 points on the screen.

## Appendix

The current eye tracker software was written in Visual C++ 8.0 (but newer versions are available). The header files and libraries are available from https://www.theimagingsource.de/produkte/software/software-development-kits-sdks/ic-imaging-control/ as well as the camera drivers.

First, the geometrical variables of the eye tracker set-up must be defined:

- screen resolution (here 1920x1080 Pix),
- video magnification (pix/mm, here 35.5),
- distance of subject to screen (550 mm),
- horizontal distance between cameras (here 80 mm),
- distance from the camera to LEDs (here 80 mm),
- distance from the camera to the eye (here 250 mm).

Based on these numbers, visual angles can be determined by simple geometry (which is automatically done by the software).

Other variables that are defined in the source code is the number of averaged eye positions when fixation was assumed because the running standard deviation of 25 eye positions dropped below 0.5 degrees (here: 100), as well as the threshold for blink detection (here 0.2 which means that the running standard deviation of pupil sizes should be less than 0.2 mm). If it passes the threshold, the measured pupil size decreased faster than naturally possible, indicating a blink. In this case, data is set to zero but the time axis of data writing continues.

Important variables that can be adjusted by the arrow keys of the keyboard are thresholds for pupil detection and for Purkinje image detection. The pupil detection threshold factor is set by default to 0.6, which means all pixels that are darker than 0.6 of the average pixel brightness of the video frame are attributed to the pupil which appears black in the video image. The pixels in the Purkinje image are typically close to saturation, and the pixel threshold for their detection is set to 250, independently from the average brightness of the video image. We have also included a simple focus detection algorithm, counting the number of pixels in the Purkinje image. The size of the Purkinje image is determined by the size and distance of the IR LED field that generates it, and also of defocus. The threshold is set to 400 pixels. If the Purkinje image is larger than that, significant defocus is present and the distance of the subject from the camera is out of range. This condition affects video magnification and therefore the measured Hirschberg ratio. However, since the eye tracker needs to be used with a chin rest, the defocus detector was rarely activated during our measurements.

The software uses a global frame counter of all grabbed frames which is necessary for many timing issues. The software also regularly accesses the computer clock to determine the frame rate, simply calculated from the time used to process 30 frames and displays the number of frames/sec on the screen. The major time limiting factor is the display of graphics. It can slow the frame rate from 450 to 300 fps. Therefore, little graphics is shown during measurements after calibration of the eye tracker so that full camera speed is available as listed with the description of the camera on the home page of The Imaging Source.

At the end of a measurement session, data can be saved to a file which includes:

- all calibration parameters,
- frame number,
- time determined from frame rate,
- pupil diameters,
- eye positions in screen coordinates (in floating point pixel coordinates) in x and y direction,
- vergence determined from eye positions and after automated correction for pupil centration artifacts (in arcmin),
- the timing of a trigger signal that is linked to the appearance of a new fixation target and was used in the current study to synchronize our eye tracker to the EyeLink 1000 Plus for comparison.

Both camera inputs (Y800, monochrome, each 640x480 pixels) are sequentially loaded into one frame buffer of 1280x480 pixels. Pupil and Purkinje image detection occur therefore at the same time and not alternatingly. First, the buffer is analyzed in the left half (representing the right eye). The pupil is detected simply by collecting all pixel that are darker than average frame brightness*pupil threshold factor (0.6 as default). Coordinates of the detected pixels are stored, and the “center of mass” determined in x and y direction. Since more than 20,000 pixels are in the pupil (video magnification about 35/mm), the center of mass is located at subpixel resolution, typically by a half or less pixel resolution (equivalent to about 10 µm). The radius of the pupil can be simply determined from pupil area, assuming that the pupil is round. Similarly, the first Purkinje image is located by counting pixels brighter than 250 and determining the center of mass. In the case, only about 300 pixels are available (depending on the size of the IR LED field and its distance but the location of the center of mass nevertheless had a similar resolution as pupil center of about 10 µm). All these variables are continuously displayed on the screen during calibration, providing a clear message about the resolution of the eye tracker. The same procedures are then repeated in the right half of the frame buffer, showing the left eye. Horizontal and vertical eye positions are simply determined from the horizontal and vertical distances of the pupil centers to the Purkinje images although it has to be kept in mind that neither Kappas nor Hirschberg ratios of the eyes are known at this time and that rather the pupil axis is measured. However, both variables can be determined when it is known that the subject fixates a target on the screen with known position. Therefore, for calibration, the system must recognize when the subject fixates a target on the screen. A simple procedure is to analyze the running standard deviation of 25 subsequent eye positions. To determine the running standard deviation, 25 eye position data have to be stored backwards in an array and the standard deviation of the data is calculated for each running frame. Running standard deviation are also determined for pupil center positions, number of pixels in the pupil, Purkinje image positions, number of pixels in the Purkinje image and for absolute differences of subsequent measurements. These standard deviations are all providing information about the noise level of the eye tracker and are continuously shown on the screen, together with all options and instructions for the subsequent calibration.

The calibration procedure itself starts with presentation of a red fixation spot on the screen. Typically, the subject fixates this point. To achieve better resolution, standard deviations of 100 eye positions are now tracked, rather than 25. If the standard deviation for 100 measurements of distance pupil center to Purkinje image center drops below 1 pixel, a sound signal is emitted and the red fixation spot turns green. The distances between pupil center and Purkinje image are stored for fixation point 1, and the procedure is repeated with 3 more fixation points, arranged in a rectangle with adjustable size. Finally, a fixation spot appears in the center of the rectangle but in this case, the linearity of the eye tracking procedure is tested since the measured fixation should match the position of the center point. After the calibration procedure is completed (about 2-3 sec), any eye position within the rectangular field can be inferred by linear extrapolation. At this point, it is necessary to consider how linearly the distance between pupil center and Purkinje image center are related to the true eye position. Fortunately, classical measurements (17) and our own experience show that it does not pay off to add more fixation spots and generate a two-dimensional polynomial fit of the conversion from measured pupil center and Purkinje image center data to eye position. It is more important to determine these variables very precisely when the subject fixates, and this is why 100 eye position data are averaged. With a frame rate above 400 fps, the fixation period needs to be only a fraction of a second. In practice, it is necessary to stop data collection for one fixation spot as soon as one fixation episode was successful. For this reason, the software does not collect further data after fixation was successful for a period on 500 frames (about 1 sec). The screen output during calibration is shown in Figure 1A.

Once the calibration is complete, the screen is cleared with a pixel gray value of 127, and eye tracking can start. A few features are tested:

1. Linearity of calibration. The distances between pupil center and the center of the first Purkinje image are shown, normalized to the center where they are on top of each other (Figure 2A). This plot shows potential distortions in the calibration map and lists all variables used for calibration. It also shows how the calibration procedure optimizes the orthogonality (red, before calibration, yellow after calibration).

2. A green fixation point is shown in the center of the screen. The screen turns black (0) for about 3 sec and then becomes bright (255) for 30 frames, about 1/10 sec. This elicits a pupil response. The software plots the measured convergence of both eyes versus pupil size. If the pupil center position is not stationary but rather moves when pupil size changes, a correction for pupil size changes becomes necessary for future eye tracking. To visualize that this is necessary, the software plots pupil sizes versus convergence and performs a linear regression. If the slope is significant from zero, a correction is necessary and implanted in the recorded data which are now dependent on pupil size (Figure 3A). During this step, also Hirschberg ratios and Kappas for both eyes are determined and shown on the screen.

A few fixation points appear on the screen, one after the other and presented together with a sound signal, and the eye tracker records eye position during fixation. Subsequently, all fixation data are plotted on the screen on top of the fixation points and allow a rapid evaluation of the quality of measurements (Figure 4A).

Now eye tracking measurements can start. The user has the options to either write the fixation axes of both eyes back on the screen, present pictures on the screen, either gaze-contingently (left or right eye) or stationary, or even present stereo gaze-contingently for each eye at the same time, using red-green spectacles and red-green images.

During the measurements, all data are continuously written as ASCII data to a file at 300-400 Hz for offline analysis.

We attach the software for our eye tracker:

- a video showing the procedures can be downloaded here: https://www.dropbox.com/s/7k6c6h37nljzl3i/DEMO%20eye%20tracker%20Feb%202021.wmv?dl=0
- the software of the eye tracker, with libraries, camera drivers and IC Imaging Control 3.1: https://www.dropbox.com/sh/kpejv5p8ud6bxwl/AABRs6-950UOxUU2FmA8Er0ya?dl=0
- instructions for the eye tracker set-up: https://www.dropbox.com/s/e8dck6ld6hg91v6/Instructions%20binocular%20eye%20tracker.pdf?dl=0 .

## References

[b11] Baden, T., Maina, M. B., Maia Chagas, A., Mohammed, Y. G., Auer, T. O., Silbering, A., von Tobel, L., Pertin, M., Hartig, R., Aleksic, J., Akinrinade, I., Awadelkareem, M. A., Koumoundourou, A., Jones, A., Arieti, F., Beale, A., Münch, D., Salek, S. C., Yusuf, S., & Prieto-Godino, L. L. (2020, 8 5). TReND in Africa: Toward a Truly Global (Neuro)science Community. Neuron, 107(3), 412–416. 10.1016/j.neuron.2020.06.026 1097-4199 32692973 PMC7370902

[b16] Barsingerhorn, A. D., Boonstra, F. N., & Goossens, H. H. (2017, 1 9). Optics of the human cornea influence the accuracy of stereo eye-tracking methods: A simulation study. Biomedical Optics Express, 8(2), 712–725. 10.1364/BOE.8.000712 2156-7085 28270978 PMC5330588

[b19] Bartl, K., Siebold, C., Glasauer, S., Helmchen, C., & Büttner, U. (1996, 4). A simplified calibration method for three-dimensional eye movement recordings using search-coils. Vision Research, 36(7), 997–1006. 10.1016/0042-6989(95)00201-4 0042-6989 8736259

[b37] Bellet, M. E., Bellet, J., Nienborg, H., Hafed, Z. M., & Berens, P. (2019, 2 1). Human-level saccade detection performance using deep neural networks. Journal of Neurophysiology, 121(2), 646–661. 10.1152/jn.00601.2018 1522-1598 30565968

[b24] Brand, J., Diamond, S. G., Thomas, N., & Gilbert-Diamond, D. (2020, 11 27). Evaluating the data quality of the Gazepoint GP3 low-cost eye tracker when used independently by study participants. Behavior Research Methods, Advance online publication. 10.3758/s13428-020-01504-2 1554-3528 33245514

[b17] Brodie, S. E. (1987, 4). Photographic calibration of the Hirschberg test. Investigative Ophthalmology & Visual Science, 28(4), 736–742.0146-0404 3557878

[b1] Carr DB, Grover P. The Role of Eye Tracking Technology in Assessing Older Driver Safety. Geriatrics (Basel). 2020 6 7;5(2):36. doi: 10.3390/geriatrics5020036. PMID: 32517336; PMC-ID: PMC7345272. PMC7345272 32517336

[b34] Chen, C. Y., & Hafed, Z. M. (2013, 3 20). Postmicrosaccadic enhancement of slow eye movements. The Journal of Neuroscience : The Official Journal of the Society for Neuroscience, 33(12), 5375–5386. 10.1523/JNEUROSCI.3703-12.2013 1529-2401 23516303 PMC6704992

[b13] Cornsweet, T. N., & Crane, H. D. (1973, 8). Accurate two-dimensional eye tracker using first and fourth Purkinje images. Journal of the Optical Society of America, 63(8), 921–928. 10.1364/josa.63.000921 10.1364/josa.63.000921 0030-3941 4722578

[b14] Crane, H. D., & Steele, C. M. (1985, 2 15). Generation-V dual-Purkinje-image eyetracker. Applied Optics, 24(4), 527. 10.1364/ao.24.000527 10.1364/ao.24.000527 1559-128X 18216982

[b25] Dalmaijer, E. “Is the Low-Cost EyeTribe Eye Tracker Any Good for Research?” 2014, doi:10.7287/peerj.preprints.585v1

[b2] Frutos-Pascual, M., & Garcia-Zapirain, B. (2015, 5 12). Assessing visual attention using eye tracking sensors in intelligent cognitive therapies based on serious games. Sensors (Basel), 15(5), 11092–11117. 10.3390/s150511092 1424-8220 25985158 PMC4481919

[b38] Hafed, Z. M. (2013, 2 20). Alteration of visual perception prior to microsaccades. Neuron, 77(4), 775–786. 10.1016/j.neuron.2012.12.014 1097-4199 23439128

[b39] Hafed, Z. M., Chen, C. Y., & Tian, X. (2015, 12 2). Vision, Perception, and Attention through the Lens of Microsaccades: Mechanisms and Implications. Frontiers in Systems Neuroscience, 9, 167. 10.3389/fnsys.2015.00167 1662-5137 26696842 PMC4667031

[b36] Holmqvist, K. “Eye Tracker Data Quality.” Proceedings of the Symposium on Eye Tracking Research and Applications - ETRA '12, 2012, doi:10.1145/2168556.2168563

[b44] Hosp, B., Eivazi, S., Maurer, M., Fuhl, W., Geisler, D., & Kasneci, E. (2020, 6). RemoteEye: An open-source high-speed remote eye tracker : Implementation insights of a pupil- and glint-detection algorithm for high-speed remote eye tracking. Behavior Research Methods, 52(3), 1387–1401. 10.3758/s13428-019-01305-2 1554-3528 32212086

[b20] Houben, M. M., Goumans, J., & van der Steen, J. (2006, 1). Recording three-dimensional eye movements: Scleral search coils versus video oculography. Investigative Ophthalmology & Visual Science, 47(1), 179–187. 10.1167/iovs.05-0234 0146-0404 16384960

[b31] Hutchinson, T. E., White, K. P., Martin, W. N., Reichert, K. C., & Frey, L. A. (1989). Human-Computer Interaction Using Eye-Gaze Input. IEEE Transactions on Systems, Man, and Cybernetics, 19(6), 1527–1534. 10.1109/21.44068 0018-9472

[b21] Imai, T., Sekine, K., Hattori, K., Takeda, N., Koizuka, I., Nakamae, K., Miura, K., Fujioka, H., & Kubo, T. (2005, 3). Comparing the accuracy of video-oculography and the scleral search coil system in human eye movement analysis. Auris, Nasus, Larynx, 32(1), 3–9. 10.1016/j.anl.2004.11.009 0385-8146 15882818

[b26] Janthanasub, V. (2015). PhayungM. “Evaluation of a Low-Cost Eye Tracking System for Computer Input. KMUTNB International Journal of Applied Science and Technology., 10.14416/j.ijast.2015.07.001

[b12] Karikari, T. K., Cobham, A. E., & Ndams, I. S. (2016, 2). Building sustainable neuroscience capacity in Africa: The role of non-profit organisations. Metabolic Brain Disease, 31(1), 3–9. 10.1007/s11011-015-9687-8 1573-7365 26055077 PMC4718937

[b40] Ko, H. K., Poletti, M., & Rucci, M. (2010, 12). Microsaccades precisely relocate gaze in a high visual acuity task. Nature Neuroscience, 13(12), 1549–1553. 10.1038/nn.2663 1546-1726 21037583 PMC3058801

[b6] Letaief, M., Rezzoug, N., & Gorce, P. (2021, 1 2). Comparison between joystick- and gaze-controlled electric wheelchair during narrow doorway crossing: Feasibility study and movement analysis. Assistive Technology, 33(1), 26–37. 10.1080/10400435.2019.1586011 1949-3614 30945980

[b3] Lu, Z., Coster, X., & de Winter, J. (2017, 4). How much time do drivers need to obtain situation awareness? A laboratory-based study of automated driving. Applied Ergonomics, 60, 293–304. 10.1016/j.apergo.2016.12.003 1872-9126 28166888

[b32] Malevich, T., Buonocore, A., & Hafed, Z. M. (2020, 8 6). Rapid stimulus-driven modulation of slow ocular position drifts. eLife, 9, e57595. 10.7554/eLife.57595 2050-084X 32758358 PMC7442486

[b41] Martinez-Conde, S., Macknik, S. L., & Hubel, D. H. (2004, 3). The role of fixational eye movements in visual perception. Nature Reviews. Neuroscience, 5(3), 229–240. 10.1038/nrn1348 1471-003X 14976522

[b8] Meyer, A. F., Poort, J., O’Keefe, J., Sahani, M., & Linden, J. F. (2018, 10 10). A Head-Mounted Camera System Integrates Detailed Behavioral Monitoring with Multichannel Electrophysiology in Freely Moving Mice. Neuron, 100(1), 46–60.e7. 10.1016/j.neuron.2018.09.020 1097-4199 30308171 PMC6195680

[b27] Morgante JD, Zolfaghari R, Johnson SP. A Critical Test of Temporal and Spatial Accuracy of the Tobii T60XL Eye Tracker. Infancy. 2012 1;17(1):9-32. doi: 10.1111/j.1532-7078.2011.00089.x.Epub 2011 Aug 29. Erratum in: Infancy. 2012 Mar;17(2):245. PMID: 32693503. 10.1111/j.1532-7078.2011.00089.x.Epub 32693503

[b45] Niehorster, D. C., Zemblys, R., & Holmqvist, K. (2021, 2). Is apparent fixational drift in eye-tracking data due to filters or eyeball rotation? Behavior Research Methods, 53(1), 311–324. 10.3758/s13428-020-01414-3 1554-3528 32705655 PMC7880979

[b28] Ooms, K., Dupont, L., Lapon, L., & Popelka, S. (2015). Accuracy and Precision of Fixation Locations Recorded with the Low-Cost Eye Tribe Tracker in Different Experimental Setups. Journal of Eye Movement Research, 8(1). Advance online publication. 10.16910/jemr.8.1.5 1995-8692

[b4] Orlov, P. A., & Apraksin, N. (2015). The Effectiveness of Gaze-Contingent Control in Computer Games. Perception, 44(8-9), 1136–1145. 10.1177/0301006615594910 0301-0066 26562927

[b9] Payne, H. L., & Raymond, J. L. (2017, 9 5). Magnetic eye tracking in mice. eLife, 6, e29222. 10.7554/eLife.29222 2050-084X 28872455 PMC5584990

[b29] Raynowska, J., Rizzo, J. R., Rucker, J. C., Dai, W., Birkemeier, J., Hershowitz, J., Selesnick, I., Balcer, L. J., Galetta, S. L., & Hudson, T. (2018). Validity of low-resolution eye-tracking to assess eye movements during a rapid number naming task: Performance of the eyetribe eye tracker. Brain Injury : [BI], 32(2), 200–208. 10.1080/02699052.2017.1374469 1362-301X 29211506 PMC6028183

[b22] Robinson D. A Method Of Measuring Eye Movement Using A Scleral Search Coil In A Magnetic Field. Ieee Trans Biomed Eng. 1963 10;10:137-45. Doi: 10.1109/Tbmel.1963.4322822. Pmid: 14121113. 10.1109/Tbmel.1963.4322822 14121113

[b42] Rucci, M. (2008). Fixational eye movements, natural image statistics, and fine spatial vision. Network (Bristol, England), 19(4), 253–285. 10.1080/09548980802520992 1361-6536 18991144

[b30] Schaeffel, F. (2002, 5). Kappa and Hirschberg ratio measured with an automated video gaze tracker. Optometry and Vision Science, 79(5), 329–334. 10.1097/00006324-200205000-00013 1040-5488 12035991

[b5] Strobl, M. A. R., Lipsmeier, F., Demenescu, L. R., Gossens, C., Lindemann, M., & De Vos, M. (2019, 5 3). Look me in the eye: Evaluating the accuracy of smartphone-based eye tracking for potential application in autism spectrum disorder research. Biomedical Engineering Online, 18(1), 51. 10.1186/s12938-019-0670-1 1475-925X 31053071 PMC6499948

[b18] Tabernero, J., & Artal, P. (2014, 4 22). Lens oscillations in the human eye. Implications for post-saccadic suppression of vision. PLoS One, 9(4), e95764. 10.1371/journal.pone.0095764 1932-6203 24755771 PMC3995773

[b35] Tian, X., Yoshida, M., & Hafed, Z. M. (2016, 3 7). A Microsaccadic Account of Attentional Capture and Inhibition of Return in Posner Cueing. Frontiers in Systems Neuroscience, 10, 23. 10.3389/fnsys.2016.00023 1662-5137 27013991 PMC4779940

[b23] van der Geest, J. N., & Frens, M. A. (2002, 3 15). Recording eye movements with video-oculography and scleral search coils: A direct comparison of two methods. Journal of Neuroscience Methods, 114(2), 185–195. 10.1016/s0165-0270(01)00527-1 10.1016/s0165-0270(01)00527-1 0165-0270 11856570

[b33] Wang, D., Mulvey, F. B., Pelz, J. B., & Holmqvist, K. (2017, 6). A study of artificial eyes for the measurement of precision in eye-trackers. Behavior Research Methods, 49(3), 947–959. 10.3758/s13428-016-0755-8 1554-3528 27383751

[b15] Wildenmann, U., & Schaeffel, F. (2013, 11). Variations of pupil centration and their effects on video eye tracking. [Erratum in: Ophthalmic Physiol Opt. 2014 Jan;34] [1] [:123. PMID: 24102513]. Ophthalmic & Physiological Optics, 33(6), 634–641. 10.1111/opo.12086 1475-1313 24102513

[b43] Willeke, K. F., Tian, X., Buonocore, A., Bellet, J., Ramirez-Cardenas, A., & Hafed, Z. M. (2019, 8 16). Memory-guided microsaccades. Nature Communications, 10(1), 3710. 10.1038/s41467-019-11711-x 2041-1723 31420546 PMC6697692

[b7] Wästlund, E., Sponseller, K., Pettersson, O., & Bared, A. (2015). Evaluating gaze-driven power wheelchair with navigation support for persons with disabilities. Journal of Rehabilitation Research and Development, 52(7), 815–826. 10.1682/JRRD.2014.10.0228 1938-1352 26744901

[b10] Zoccolan, D., Graham, B. J., & Cox, D. D. (2010, 11 29). A self-calibrating, camera-based eye tracker for the recording of rodent eye movements. Frontiers in Neuroscience, 4, 193. 10.3389/fnins.2010.00193 1662-453X 21152259 PMC2998901

